# Clinical effects of unilateral biportal endoscopic decompression for lumbar posterior apophyseal ring separation

**DOI:** 10.3389/fsurg.2022.948417

**Published:** 2022-07-28

**Authors:** Jianjun Liu, Bin Zhu, Lei Chen, Juehua Jing, Dasheng Tian

**Affiliations:** The Department of Orthopedics, The Second Hospital of Anhui Medical University, Hefei, China

**Keywords:** unilateral biportal endoscopic, decompression, lumbar, posterior apophyseal ring separation, clinical effects

## Abstract

**Objective:**

The purpose of the study was to investigate the feasibility and effects of unilateral biportal endoscopic decompression for lumbar posterior apophyseal ring separation (PARS).

**Methods:**

Patients with lumbar PARS who received unilateral biportal endoscopic decompression from June 2020 to September 2021 were analyzed, including 11 females and 15 males. The clinical symptoms were consistent with the imaging findings. Operation time, length of postoperative hospital stay and complications were recorded, and the clinical efficacy was evaluated by Visual Analogue Scale (VAS), Oswestry Disability Index (ODI) and modified Macnab scale at preoperative, postoperative 1, 3, 6 months and the last follow-up.

**Results:**

Preoperative VAS scores of low back pain were (5.04 ± 1.37) and respectively decreased to (2.81 ± 0.75), (2.35 ± 0.98), (1.65 ± 0.69) and (1.15 ± 0.68) at postoperative 1, 3, 6 months and at the last follow-up, and the difference was statistically significant (F = 127.317, *P* = 0.000). Preoperative VAS scores of lower limb pain were (6.92 ± 1.38) and respectively decreased to (2.88 ± 1.07), (2.54 ± 1.03), (1.81 ± 0.80) and (1.00 ± 0.69) at postoperative 1, 3, 6 months and at the last follow-up, and the difference was statistically significant (F = 285.289, *P* = 0.000). Preoperative ODI scores were (60.47 ± 8.89) and respectively decreased to (34.72 ± 4.13), (25.80 ± 3.65), (17.71 ± 3.41) and (5.65 ± 2.22) at postoperative 1, 3, 6 months and at the last follow-up, and the difference was statistically significant (F = 725.255, *P* = 0.000). According to the modified Macnab criteria, the final outcome was excellent in 22 cases, good in 3 cases, fair in 1 cases. 26 patients could return to work or normal activities within 3 weeks.

**Conclusions:**

Unilateral biportal endoscopic decompression has the advantages of clear and wide field of vision, large operating space, relatively simple need of surgical instrument and convenient and flexible operation procedure. It can achieve excellent clinical results with favorable efficacy and safety and may become a new minimally invasive endoscopic treatment for lumbar PARS.

## Background

Lumbar posterior apophyseal ring separation (PARS) is initiated in adolescents and often accompanied by lumbar disc herniation (1). Its mechanism remains unknown and different scholars have different views. The dural sac or nerve root can be compressed by herniated disc and separated bony fragment, which leads to back pain and neural symptoms among suffers (2). The disorder gradually proceeds and seriously hampers the normal life of patients. Conservative treatment is usually not satisfactory, and most of patients need surgical treatment (3).

Most patients were treated with open surgery in the past. Although the decompression was complete, there were risks of large trauma, more bleeding, spinal instability (4). When the fusion surgery was used, such as posterior lumbar interbody fusion (PLIF) or transforaminal lumbar interbody fusion (TLIF), drawbacks of the traditional fusion surgery appeared, such as adjacent segment degeneration, failed back surgery syndrome (5). Recently, with the deepening of the concept of minimally invasive spine surgery and the development of minimally invasive spine surgery techniques, percutaneous endoscpic discectomy has been used to treat this kind of disease (6). It has the advantages of less trauma, quick recovery, and no damage to paravertebral muscles and ligament. It has little impact on spinal stability and allows early out-of-bed functional exercise and reduces the occurrence of postoperative complications (7). However, the working portal and viewing portal are coaxial and the movable range of working portal is small. Due to the obstruction of joint process, pedicle, posterior margin of vertebral body, especially obstruction of high iliac crest in the L5/S1 segment, precise targeted catheterization is difficult for pertcutaneous transforaminal endoscopic discetomy. Therefore, the satisfactory decompression of the spinal canal is a challenging process. MED uses a single working channel and has the limitations of operation flexibility, operation space and poor vision of surgical field, which can easily lead to neural damage (8). Additionally, special working portal can easily lead to muscle strain injury.

Unilateral biportal endoscopic (UBE) technique utilizes two portals to complete the decompression, which are not coaxial. Viewing portal is used to expose the surgical field with arthroscopy and continuously rinse to keep the field clear, and the working portal is used for neural decompression through the posterior interlaminar approach, which is similar to traditional posterior open surgery (9). One of the advantages of this technique is that the two percutaneous portals are separated from each other and do not interfere with each other. Without portals limitation, endoscopic and surgical instruments can be moved freely and the whole operation is convenient and flexible (10). All directions and parts of the spinal canal can be explored. This technique can not only reach the goal of minimally invasive spine surgery, but also obtain the similar decompression effect close to open surgery, which is a supplement to the existing endoscopic technology (11).

This paper summarized 26 cases with lumbar PARS who were treated with UBE technique, and discussed the application and clinical efficacy of UBE technique in the treatment of lumbar PARS.

## Materials and methods

### Patient information

A retrospective analysis was performed on 26 patients treated with UBE technique for lumbar PARS in the authors' hospital from June 2020 to September 2021. The inclusion criteria were as the following: (1) Imaging examination (CT and MRI) confirmed lumbar disc herniation with PARS, the symptoms and signs were consistent with imaging and the responsible segment was single; (2) Neurogenic claudication or radicular leg pain with or without back pain; (3) Conservative treatment is poor or recurrent attacks; (4) The patients received unilateral biportal endoscopic decompression.

Exclusion criteria were as the following: (1) segmental instability; (2) lumbar spinal stenosis; (3) lumbar spondylolisthesis; (4) surgery history of targeted segment; (5) infectious history of lumbar spine; (5) Calcified lumbar disc herniation; (6) History of mental illness.

The study was approved by our institutional review board and the informed consent was obtained from all patients.

### Surgical procedures

#### Patients preparation

All cases were performed by single surgeon. After induction of general anesthesia, patients are positioned prone with the abdomen free and the spine flexed to open the interlaminar space.

#### Placement of endoscopic portals

After level confirmation is conducted under the C-arm fluoroscopic guidance, two portals are made 1 cm parallel to midline of spinous process and 1.0 cm above and 1.0 cm below the center of the target level. The proximal portal is about 6 mm to introduce the arthroscope and the distal portal is about 10 mm to place the surgical instruments. The fascia perpendicular to the skin is incised to prevent the obstruction of water flow during surgery. The distance between both portals allows the surgeon to perform the triangulation technique with complete freedom of the surgical tool. The primary dilator is then inserted into the two portals through the paraspinal muscles without any separation till it is docked over the lamina surface and then it is uesd to separate bluntly and push aside the overlying soft tissue step by step to form a visual surgical field.

#### Insertion of the endoscope and preparation of the surgical field

**Figure 1:**
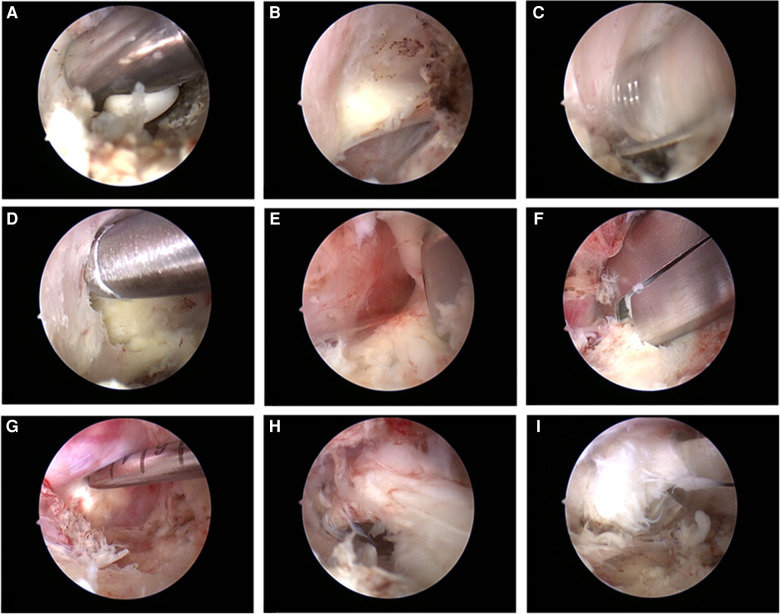
Intraoperative processes. (**A**) The radiofrequency probe is used to clean the remaining soft tissues or muscles over the lamina and ligamentum flavum; (**B**) The ligamentum flavum of the target interlaminar space and inferior edge of superior lamina are completely exposed; (**C**) The arthroscopic burr is used to thin out ispilateral lamina; (**D**) Kerrison punch is used to complete a hemilaminotomy until the upper edge of deep part of ligamentum flavum is free; (**E**) The ligament is peeled down in caucal direction and is removed using the Kerrison punch; (**F**) Kerrison punch is used to undercut the facet down to the medial wall of the pedicle; (**G**) The adhesion between the nucleus pulposus and the surrounding soft tissue is separated by a probe; (**H**) The herniated nucleus pulposus is removed by using forceps; (**I**) Kerrison punch and forceps are used to remove the separated bony fragment of vertebral body.

The endoscopic cannula and trochar are introduced through the endoscopic portal till they are docked over the superior lamina. The irrigation fluid is initiated and the trochar is removed to wash out the blood and the endoscope with 30° lens is introduced through the cannula. The irrigation fluid used is isotonic saline to avoid tissue edema. Then the radiofrequency probe is used to clean the remaining soft tissues or muscles over the lamina and ligamentum flavum (Figure 1A).

### Laminotomy and ligamentum flavum removal

When the ligamentum flavum of the target interlaminar space and inferior edge of superior lamina are completely exposed (Figure 1B), the arthroscopic burr is used to thin out ispilateral lamina (Figure 1C), which is followed by laminectomy by Kerrison punch to complete a hemilaminotomy until the upper edge of deep part of ligamentum flavum is free (Figure 1D). After ensuring that the plane between ligamentum flavum and dura is free from adhesion, the ligament is peeled down in caucal direction and is removed using the Kerrison punch (Figure 1E).

### Decompression

After identification of the nerve root adjacent to the dural sac, the spinal canal is explored according to direction of nucleus pulposus herniation. According to the needs, forceps or drill are used to enlarge lamina window. We prefer to undercut the facet down to the medial wall of the pedicle (Figure 1F). This work allows for the discectomy to be conducted with less nerve root retraction in addition to achieving lateral recess decompression. However, attention should be paid to protect the facet joint structure to avoid excessive damage to the spinal stability. After the herniated nucleus pulposus is found, the adhesion between the nucleus pulposus and the surrounding soft tissue is separated by a probe (Figure 1G). After assistant retracts dural sac or nerve root using an L-type nerve retractor through the working portal, the surgeon uses forceps to remove the herniated nucleus pulposus (Figure 1H). Annulotomy could be performed using a microknife if it is required. Then the surgeon needs to adjust the working position and explore the targeted intervertebral space. Any remnant fragments of the herniated disc need to be removed. We prefer to use Kerrison punch and forceps to remove the separated bony fragment of vertebral body (Figure 1I). The procedure is completed after conforming the complete decompression and freely movement of nerve root. It is not necessary to remove the separated bony fragment completely to avoid retracting the nerve excessively if the bony fragment don't lead to nerve tissue compression.

### Closure

The endoscope and instruments are moved and remaining fluid is discharged by squeezing the skin around the portals. A drainage tube is placed in all patients through the working portal to prevent hematoma formation, followed by wound closure.

### Outcome measures

Operation time, length of postoperative hospital stay and complications were recorded. The clinical efficacy was evaluated by Visual Analogue Scale (VAS), Oswestry Disability Index (ODI) and modified Macnab scale at preoperative, postoperative 1, 3, 6 months and the last follow-up.

### Statistical analysis

Data were statistically described in terms of mean ± standard deviation (SD), or frequencies (number of cases) and percentages when appropriate. We conducted general linear model with repeated measures to analyze the clinical efficacy before the operation and at the follow-up and we compared numerical variables between different follow-up times using Student t test. *P* values <0.05 were considered statistically significant. We used SPSS 22.0 (Statistical Package for the Social Science; SPSS Inc., Chicago, IL, USA) for statistical analysis.

## Results

### Demographic data

The patients who conform to the inclusion criteria underwent UBE technique for lumbar PARS. The study included 15 men and 11 women, with a average age of (37.27 ± 7.72) years. On the targeted levels, 7 cases were at L4/5, and 19 cases were at L5/S1.

### Surgical technique-related outcome

All patients were followed up for more than 6 months, with an average of (13.27 ± 3.96) months. The operative time was (78.27 ± 18.58) minutes. The postoperative hospital stay was (4.58 ± 1.42) d.

### Clinical outcomes

VAS scores of low back pain were improved after operation Preoperative VAS scores of low back pain were (5.04 ± 1.37) and respectively decreased to (2.81 ± 0.75), (2.35 ± 0.98), (1.65 ± 0.69) and (1.15 ± 0.68) at postoperative 1, 3, 6 months and at the last follow-up, and the difference was statistically significant (F = 127.317, *P* = 0.000). The VAS scores of lower limb pain were improved after operation. Preoperative VAS scores of lower limb pain were (6.92 ± 1.38) and respectively decreased to (2.88 ± 1.07), (2.54 ± 1.03), (1.81 ± 0.80) and (1.00 ± 0.69) at postoperative 1, 3, 6 months and at the last follow-up, and the difference was statistically significant (F = 285.289, *P* = 0.000). ODI scores were improved after operation. Preoperative ODI scores were (60.47 ± 8.89) and respectively decreased to (34.72 ± 4.13), (25.80 ± 3.65), (17.71 ± 3.41) and (5.65 ± 2.22) at postoperative 1, 3, 6 months and at the last follow-up, and the difference was statistically significant (F = 725.255, *P* = 0.000) (Table 1). According to the modified Macnab criteria, the final outcomes were excellent in 22 cases, good in 3 cases, fair in 1 case at the final follow-up, with an excellent-or-good rate of 96.2% (25/26).

**Table 1:** Clinical outcomes in different times.

Time	VAS scores (back pain)	VAS scores (lower limb pain)	ODI (%)
Preoperative	5.04 ± 1.37	6.92 ± 1.38	60.47 ± 8.89
Postoperative 1 month	2.81 ± 0.75	2.88 ± 1.07	34.72 ± 4.13
Postoperative 3 month	2.35 ± 0.98	2.54 ± 1.03	25.80 ± 3.65
Postoperative 6 month	1.65 ± 0.69	1.81 ± 0.80	17.71 ± 3.41
Final follow-up	1.15 ± 0.68	1.00 ± 0.69	5.65 ± 2.22
*P* value	F = 127.317, *P *= 0.000	F = 285.289, *P = *0.000	F = 725.255, *P = *0.000

Values are presented as mean ± standard deviation.

P < 0.05 considered as significant.

### Complications

**Figure 2:**
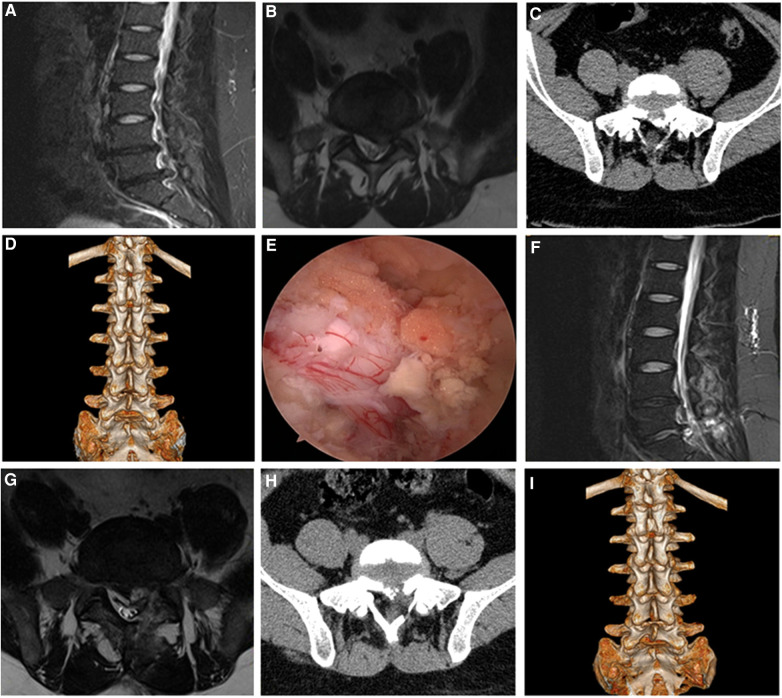
Male, 28 years old, L5/S1 lumbar disc herniation with posterior apophyseal ring separation. (**A**) Preoperative sagittal MR image showed L5/S1 lumbar disc herniation; (**B**) Preoperative axial MR image showed herniated lumbar disc compressed nerve root and dural sac; (**C**) Preoperative axial CT image showed separated bony fragment of vertebral body; (**D**) Preoperative 3D-CT image; (**E**) Intraoperative image after complete neural decompression; (**F**) Postoperative sagittal MR image revealed the complete decompression of the spinal canal; (**G**) Postoperative axial MR image showed the complete removal of herniated disc and bony fragment; (**H**) Postoperative axial CT image showed the removal of the bony fragment; (**I**) Postoperative 3D-CT image showed the lamina window and preservation of the facet joints.

**Figure 3:**
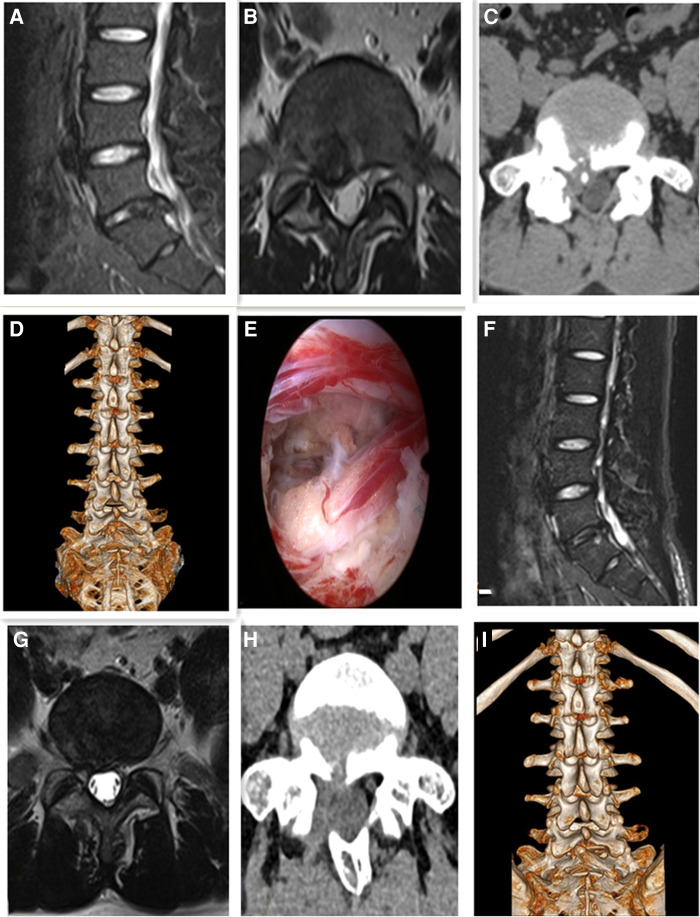
Male, 25 years old, L5/S1 lumbar disc herniation with posterior apophyseal ring separation. (**A**) Preoperative sagittal MR image showed L5/S1 lumbar disc herniation; (**B**) Preoperative axial MR image showed herniated lumbar disc compressed nerve root and dural sac; (**C**) Preoperative axial CT image showed separated bony fragment of vertebral body; (**D**) Preoperative 3D-CT image; (**E**) Intraoperative image after complete neural decompression; (**F**) Postoperative sagittal MR image revealed the complete decompression of the spinal canal; (**G**) Postoperative axial MR image showed the complete removal of herniated disc and bony fragment; (**H**) Postoperative axial CT image showed the removal of the posterior bony fragment; (**I**) Postoperative 3D-CT image showed the lamina window and preservation of the facet joints.

Intraoperative dural tear occurred in 1 case. Since the breach was very small, so we didn't repair the dural sac tears. No cerebrospinal fluid leakage occurred after the operation, and no discomfort symptoms occurred after the operation. No serious complications, such as vascular and nerve injury occurred after operation. Typical cases were shown in the Figures 2, 3.

## Discussions

Lumbar posterior apophyseal ring separation is often accompanied by lumbar disc herniation and lumbar spinal canal or lateral recess stenosis, which can cause corresponding radicular symptoms or syndrome of cauda equina. Conservative treatment is usually not effective and patients with symptoms of nerve injury need surgical treatment as soon as possible. Traditional posterior open surgery is generally considered as the standard treatment, including fenestration, hemilaminectomy, total laminectomy and fusion. Although the decompression of open surgery is complete, there are risks of large surgical trauma, excessive bleeding and spinal instability. The fusion surgery also has shortcomings, such as adjacent segment degeneration, failed back surgery syndrome.

With the development of minimally invasive concept, minimally invasive spine surgery has gradually become the mainstream. It is effective to achieve complete neurological decompression and improve clinical symptoms and quality of patients' life without affecting the stability of the lumbar spine. UBE technique achieves adequate neural decompression through posterior interlaminar approach and its principle is similar to extended interlaminar fenestration surgery. The technique uses two portals to complete the decompression. The viewing portal is used to place the endoscope with continuous irrigation, and the working portal is used to complete decompression. The absence of a common working portal for the endoscope and instruments allows for independent movement and angulation of the surgical tool, which markedly reduces the procedure's difficulty. The surgical field of the UBE technique is similar to traditional open surgery, and intraoperative procedure is more similar to open surgery. So UBE technique has a relatively easy learning curve once the surgeon gets accustomed to triangulation technique (12). UBE technique reduces the incidence of complications such as nerve injury, dural sac injury because the operation is under direct vision. The technique can use ordinary spine instruments and move them freely through the working portal. UBE technique generally uses arthroscope as endoscope and structures under the contralateral lamina can be easily observed by a 30° endoscopic lens (13). The decompression is sufficient and effective and it has a unique advantage for decompression of spinal stenosis compared with other endoscopic technique (14). Also, the continuous irrigation serves in creating a potential working space and the water pressure created inhibits the epidural bleeding. UBE technique utilizes two portals to complete the decompression and can avoid the shortcomings occurred to traditional open surgery, including large surgical trauma, excessive bleeding, spinal instability and failed back surgery syndrome.

Our study mainly investigated the feasibility and effect of unilateral biportal endoscopic decompression for lumbar PARS. All patients successfully received complete neural decompression. VAS scores of low back pain and lower limb pain were improved after operation and remained good during the follow-up period. ODI scores were improved after operation and remained good during the follow-up period. These results showed that UBE technique could achieve good clinical effects for treatment of lumbar PARS.

It's found that lumbar PARS is often accompanied by intervertebral disc herniation. The three-dimensional reconstruction of CT is valuable in the evaluation of size, shape and position of posterior bony fragment of the vertebral body, so it's the best auxiliary method for the evaluation of posterior edge (15). MRI can further show the scope of decompression during the operation (5, 16). It is important for determine the size, position and type of posterior edge before operation and whether the bony fragment behind the vertebral body is removed or not is the key and difficult point in the treatment of lumbar PARS. It still remains controversial whether the separated bony fragment should be removed simultaneously when the decompression and discectomy are done. Some authors thought that the removal of disc alone was not sufficient enough to relieve nerve compression because the bony fragment occupied the spinal space to a certain extent and triggered the symptoms more severely. They advocated the removal of the bony fragment when the decompression and discectomy were performed and reported that their clinical effects were satisfactory (17, 18). However, some authors thought that discectomy and decompression were enough (19, 20). Akhaddar et al. also supported this view and divided PARS into type I (with immobile bony fragment) /type II (with mobile fragment) and Stage A/B. They found that it was the existence of herniated disc in Type I PARS that triggered acute typical sciatica rather than the separated bony fragment, especially in Stage B and thus the removal of detached bony fragment was not necessary. On the contrary, the mobile bony fragment must be removed in Type II PARS, because the unstable bony fragment could be displaced and might damage neural structures and the clinical results were satisfactory without removal of bony fragments in 55 patients with PARS in the study (6).

In our study, the bony fragment was removed if it was not connected to vertebral body, whether it led to nerve compression or not. While the bony fragment was connected to vertebral body, it did not need to be removed if it did not lead to nerve compression and the herniated nucleus pulposus should be removed completely; however, the bony fragment needed to be removed if it led to nerve root compression. After discectomy, the tension of the nerve root should be examined to see whether the compression still existed as a result of the bony fragment. Because the bony fragment is less pliable that the disc, the safe removal is of great challenge and technical manipulation. UBE technique uses two portals to complete the decompression and allows for independent movement and angulation of the surgical tool being unrestricted by the endoscope. The surgical field of the UBE technique is similar to traditional open surgery and the use of 30° endoscopic lens can easily achieve structures under the contralateral lamina. So the UBE technique can remove the bony fragment safely with reduction in the incidence of complications and the follow-up results certified it. However, there are disadvantages in the UBE technique for treatment of lumbar PARS. The surgeon needs training on baisc arthroscopic triangulation technique to master the biportal approach. UBE technique may be more invasive than percutaneous endoscopic lumbar discectomy for the treatment of lumbar PARS.

It is important for determine the size, position and type of posterior bony fragment before operation. For lateral type of lesions, unilateral decompression is conducted. While the lesions locates centrally or wider base-abroad, bilateral decompression is required. The bony fragment is removed in *en bloc* or in a piecemeal resection fashion with the use of curette, microdrill, or osteotome and Kerrison punch if necessary. In a word, the reasonable surgical plan should be made after systematic consideration according to stability, size, location of the fragment or its contributions to neurologic symptoms.

There are some limitations to our study. Firstly, this is not a multi-centered study and the size of the sample is small. Secondly, this is a retrospective study and lacks of randomized control group. Thirdly, the study still needs long-term follow up to further evaluate the clinical effects. Therefore, randomized control trials with long-term follow-up are needed to investigate the clinical benefits, especially the multi-centered study.

## Conclusion

Unilateral biportal endoscopic decompression has the advantages of clear and wide field of vision, large operating space, relatively simple need of surgical instrument and convenient and flexible operation procedure. It can achieve excellent clinical effects with favorable efficacy and safety and may become an alternative minimally invasive endoscopic method for treating lumbar PARS.

## Data Availability

The raw data supporting the conclusions of this article will be made available by the authors, without undue reservation.
